# Supplementary material for: Work engagement and its association with emotional intelligence and demographic characteristics among nurses in Palestinian neonatal intensive care units
Source: PLoS One. 2025 Sep 19;20(9):e0332908. doi: 10.1371/journal.pone.0332908 (PMC12448998; doi:10.1371/journal.pone.0332908)
Supplement: Attachment — Submitted filename: Response to Reviewers.pdf [file pone.0332908.s002.pdf]

Point-by-Point Response Table

Reviewer	Comment #	Item	Comment	Response	Location in Manuscript
**Editor**	1	Style Requirements	Ensure manuscript meets PLOS ONE's style requirements, including file naming.	Thank you. The manuscript has been formatted to comply with PLOS ONE's style guide, and files are named appropriately.	Entire manuscript
**Editor**	2	Data Sharing Policy	Address data sharing: explain restrictions or provide repository links.	Thank you. The Data Availability Statement clarifies ethical restrictions and provides contact details for access. No public repository is feasible due to privacy concerns.	Data Availability Statement (p. 25)
**Reviewer 1**	1	Abstract Methods	Add year of study, sampling methods, and data analysis methods.	Added: Study period (February-April 2025), convenience sampling, and analysis methods (SPSS v26, Pearson's correlation, regression).	Abstract, Methods section (p. 3)
**Reviewer 1**	2	Abstract Conclusion	Specify proposals for improving conditions and use of findings.	Added: Recommendations for EI training, mentorship programs, and gender-sensitive policies.	Abstract, Conclusion (p. 4)
**Reviewer 1**	3	Introduction	Define research problem, magnitude, necessity, and practical purpose.	Addressed: Problem defined (NICU challenges in Palestine). Magnitude explained (impact on patient outcomes). Necessity justified (gap in conflict-affected regions). Practical purpose stated (evidence-based interventions).	Introduction (pp. 5-6)
**Reviewer 1**	4	Methods	Report details on scoring/validation of study tools (SSEIT, UWES).	Added: Cronbach's alpha values, scoring ranges, and references to validation studies for both tools.	Methods (pp. 10-11)
**Reviewer 1**	5	Discussion	Compare results with other studies and integrate requested references.	Added: Comparisons with Al Btoush (Jordan), Sawalma (Palestine), and cited requested references (Mohammadi, Khademipour, Hadian).	Discussion (pp. 16-18) Introduction (p.3)

**Reviewer 1**	6	Conclusion	Add suggestions for future studies.	Added: Longitudinal EI interventions, cross-cultural comparisons, and mixed-methods research.	Conclusion (p. 22)
**Reviewer 2**	1	Proofreading	Paper needs editing.	Thank you. The manuscript has been professionally proofread and polished. Minor typo in Table 3 ("[p b.r]" → "r") corrected.	Table 3 (p. 14)
**Reviewer 2**	2	Abstract	Add rationale and clarify study design.	Added rationale ("critical need to understand factors...") and specified design ("cross-sectional, descriptive correlational").	Abstract (p. 3)
**Reviewer 2**	3	Introduction	Correlate study variables, clarify significance, avoid literature review style, define "EI" upfront.	Addressed: EI-engagement framework added. Significance linked to patient outcomes. Focused on gaps (not literature review). "EI" defined in full at first use.	Introduction (pp. 5-6)
**Reviewer 2**	4	Methods	Justify exclusion of private hospitals, add references for governmental hospitals, explain convenience sampling, sample size increase, 6-month criterion, and reliability of UWES.	Addressed: Private hospitals excluded due to resource/policy differences. Palestinian Ministry of Health reference added. Convenience sampling limitations acknowledged. Sample size increased to 230 for non-response adjustment. 6-month criterion justified (orientation period). UWES reliability ($\alpha = 0.90$) reported.	Methods (pp. 9-11)
**Reviewer 2**	5	Results	Remove response rate repetition, check "corrected from 33-65??", add regression assumptions, and interpret variance.	Addressed: Duplicate response rate removed. Regression assumptions (linearity, homoscedasticity, VIF) confirmed. Variance interpretation added ($R^2 = 0.565$).	Results (pp. 12-15)
**Reviewer 2**	6	Discussion	Provide depth on conflict-area context and cultural factors (e.g., Palestine).	Added: Cultural resilience ("sumud"), familial norms, and political instability impacts.	Discussion (pp. 18-19)

**Reviewer 2**	7	Implications	Focus on significant results and future studies.	Addressed: Prioritized EI training, shift policies, and gender disparities. Future studies suggested.	Implications (p. 22)
**Reviewer 2**	8	Table 3 (Regression)	Check assumptions, add detailed results (coefficients, t-tests), and mark significance.	Addressed: Assumptions confirmed (p. 13). Full regression results (B, Beta, t-test, p-values, CI) in Table 4. Significance marked with asterisks.	Tables 3-4 (pp. 14-15)
