# Supplementary material for: Work engagement and its association with emotional intelligence and demographic characteristics among nurses in Palestinian neonatal intensive care units
Source: PLoS One. 2025 Sep 19;20(9):e0332908. doi: 10.1371/journal.pone.0332908 (PMC12448998; doi:10.1371/journal.pone.0332908)
Supplement: Attachment — Submitted filename: Response_to_Reviewers_auresp_2.pdf [file pone.0332908.s003.pdf]

Complete Point-by-Point Response to Editor Comments

Comment #	Item	Editor's Comment	Author Response	Location in Manuscript
1	Abstract consistency	Ensure consistency between Abstract (207 nurses) and Methods (230 invited, 90.2% response rate).	Updated Abstract: "Of 230 nurses invited, 207 completed the survey [response rate = 90.2%]." Added exclusion details to Methods and Figure 1.	Abstract (p. 3), Methods (p. 7), Figure 1 (p. 8)
2	Sampling bias	Discuss limitations of convenience sampling (voluntary participation, private hospital exclusion).	Added to Limitations: "Convenience sampling may underrepresent nurses with lower engagement. Exclusion of private hospitals limits generalizability." Described mitigation strategies (high response rate, standardized protocols).	Limitations (p. 22), Methods (p. 7)
3	Causal language	Avoid causal claims ("predictors of engagement") due to cross-sectional design.	Replaced with "factors associated with" throughout (Abstract, Results, Discussion).	Abstract (p. 3), Results (p. 14), Discussion (p. 18)
4	Non-significant results	Experience (B = −0.298, *p* = .061) should not be discussed as meaningful.	Revised narrative to state experience was "not significant" (Table 4, Discussion). Removed emphasis in Implications.	Results (Table 4, p. 16), Discussion (p. 19)
5	Standardized coefficients	Report β values alongside B in regression.	Added β to Table 4 (e.g., "EI: β = 0.593").	Results (Table 4, p. 16)
6	p-value formatting	Use *p* < .001 (not *p* = .000) in Tables 3–4.	Corrected in all tables.	Tables 3–4 (pp. 15–16)
7	Theoretical framework	Explicitly name Self-Determination Theory (SDT) and link hypotheses to it.	Added SDT framework (Introduction) and hypotheses (H1–H3).	Introduction (pp. 5–6)
8	Novelty	Highlight uniqueness of Palestinian NICU context vs. other settings.	Expanded Discussion: "Political instability and resource constraints amplify EI's role in Palestinian NICUs" (p. 18).	Discussion (p. 18)

9	EI training recommendations	Provide concrete suggestions (e.g., curriculum content, delivery mode).	Added: "EI training should integrate role-playing for emotion utilization and mentorship for age-related disparities" (p. 21).	Discussion (p. 21)
10	Rotational shifts	Explain unexpected positive association (e.g., unmeasured factors like shift length).	Added: "Rotating shifts may enhance skill variety in understaffed settings" (p. 20). Suggested future research on shift length/staffing ratios.	Discussion (p. 20)
11	Ethical statement	Confirm GDPR-compliant de-identification/storage protocols.	Added: "Data were de-identified and stored in password-protected databases per GDPR standards."	Ethical Considerations (p. 12)
12	Keywords	Add "response rate," "cross-sectional," "convenience sampling."	Updated Keywords (p. 4).	Keywords (p. 4)
13	Terminology consistency	Use "rotating shift" (not "rotational") uniformly.	Standardized to "rotating shift" throughout.	Tables 1–4 (pp. 14–16), Text
14	EI subdimensions	Standardize naming (e.g., "utilizing emotions" vs. "utilization").	Used "utilizing emotions" consistently.	Results (Table 2, p. 15), Text
15	Flowchart	Add Figure 1 to illustrate recruitment/exclusions.	Added flowchart (Figure 1, p. 8).	Figure 1 (p. 8)
16	Sentence structure	Split lengthy sentences (e.g., Introduction L45–50).	Divided into two sentences: "NICUs require intense emotional labor... This study examines EI's role in..."	Introduction (p. 5)
17	Typos	Fix hyphenation (e.g., "gender-sensitive").	Corrected to "gender-sensitive" throughout.	Discussion (p. 21)
18	Demographic variables	Justify limiting to 4 variables (Reviewer #3).	Added: "Demographics were selected based on prior literature; income/geography excluded due to cultural sensitivities."	Methods (p. 10)
19	Instrument translation	Clarify if SSEIT/UWES were translated (Reviewer #3).	Noted: "Questionnaires were administered in English; non-proficient nurses excluded."	Methods (p. 10)

20	Self-administered rationale	Justify not using interviews (Reviewer #3).	Added: "Self-administered surveys reduced interviewer bias and allowed completion during shifts."	Methods (p. 10)
21	Table titles	Complete titles (population, location, period).	Revised (e.g., "Table 1. Demographic characteristics of Palestinian NICU nurses [N=207], February–April 2025").	Tables 1–3 (pp. 14–16)
22	Statistical error	Replace Pearson’s r for categorical variables with t-tests/ANOVA.	Corrected in Table 3: "t-tests/ANOVA used for categorical variables."	Results (Table 3, p. 15)
23	Variable selection method	Specify regression method (enter/stepwise).	Added: "Enter method was used for regression."	Methods (p. 13)
24	Grammar	Fix "analysis was" → "analyses were."	Corrected (p. 13).	Results (p. 13)

Complete Point-by-Point Response to Reviewers Comments

Reviewer	Comment #	Item	Comment	Response	Location in Manuscript
**Reviewer 3**	1	Sampling Representativeness	Justify convenience sampling and coverage of all government hospitals.	Clarified in Methods: "12 NICUs represent all governmental hospitals in the West Bank... Convenience sampling was used due to logistical constraints."	Methods (p. 7)
**Reviewer 3**	2	Instrument Translation	Clarify if SSEIT/UWES were translated and whether non-English-proficient nurses were excluded.	Added: "Nurses not proficient in English were excluded, as questionnaires were administered in English." Noted prior validation in Palestinian contexts.	Methods (p. 10)
**Reviewer 3**	3	Table Titles	Complete titles for Tables 1–3 (include population, location, period).	Revised table titles (e.g., "Demographic characteristics of Palestinian NICU nurses [N=207], February–April 2025").	Tables 1–3 (pp. 14–16)

**Reviewer 3**	4	Statistical Analysis	Correct Pearson correlation misuse for categorical variables; use t-tests/ANOVA.	Replaced Pearson's r for categorical variables with t-tests/ANOVA. Added note in Table 3: "Independent t-tests/ANOVA used for categorical variables."	Results (Table 3, p. 15), Methods (p. 13)
**Reviewer 3**	5	Regression Variable Selection	Specify method (enter/stepwise) and include EI in Abstract results.	Added: "The enter method was used for regression." Updated Abstract to highlight EI as the strongest predictor.	Methods (p. 13), Abstract (p. 3)
**Reviewer 3**	6	Discussion Repetition	Avoid repeating statistical results; compare with prior studies.	Restructured Discussion to focus on comparisons (e.g., EI scores vs. international studies; p. 18) and removed redundant statistics.	Discussion (pp. 18–20)
**Reviewer 3**	7	EI Score Interpretation	Contextualize EI score (117.6) relative to other studies and sociopolitical factors.	Added: "The EI score of 117.6 aligns with international norms but may reflect resilience unique to Palestinian nurses."	Discussion (p. 18)
**Reviewer 5**	1	Causation vs. Association	Rephrase implications/conclusions to avoid causal claims.	Revised Conclusion: "EI is associated with engagement" (not "serves as a protective factor").	Conclusion (p. 24)
**Reviewer 5**	2	Limitations	Add limitations (e.g., exclusion of part-time nurses, language barriers).	Expanded Limitations section (p. 22) to address selection bias, language exclusions, and private hospital exclusion.	Limitations (p. 22)
**Reviewer 5**	3	Abstract Clarity	Delete "rationale for the study" sentence; define work engagement earlier.	Removed first sentence of Abstract. Defined work engagement in Introduction's opening paragraph (p. 5).	Abstract (p. 3), Introduction (p. 5)
**Reviewer 5**	4	Shift Patterns	Clarify that shifts are not a demographic characteristic.	Removed "shift patterns" from demographic brackets in Introduction (p. 6).	Introduction (p. 6)

**Reviewer 5**	5	Hospital Inclusion Criteria	Detail inclusion criteria for hospitals (e.g., only governmental).	Added: "Only governmental hospitals were included to standardize organizational policies."	Methods (p. 7)
**Reviewer 5**	6	Grammar Correction	Fix "analysis was" to "analyses were" (plural).	Corrected to "analyses were" (p. 13).	Results (p. 13)
