# Supplementary material for: Work engagement and its association with emotional intelligence and demographic characteristics among nurses in Palestinian neonatal intensive care units
Source: PLoS One. 2025 Sep 19;20(9):e0332908. doi: 10.1371/journal.pone.0332908 (PMC12448998; doi:10.1371/journal.pone.0332908)
Supplement: Attachment — Submitted filename: Response_to_Reviewers_auresp_3.pdf [file pone.0332908.s004.pdf]

Complete Point-by-Point Response to Editor and Reviewer Comments

Comment #	Item	Editor's / Reviewer's Comment	Author Response	Location in Manuscript
**1**	**Bivariate Analyses (Table 3)**	**Reviewer #3:** Re-analyze categorical predictors (gender, shift, education) using correct tests (t-tests, ANOVA). Report full statistics (n, M, SD, t, df, p, Cohen's d, F, η², post-hoc tests). Replace Table 3 and adjust Results text.	We have completely re-analyzed the bivariate statistics as requested. Independent t-tests were used for gender and shift work. A one-way ANOVA with Tukey HSD post-hoc tests was used for educational level. Pearson's correlation is now used only for continuous variables (age, experience, EI). The new Table 3 presents all requested statistics. The Results text has been revised to accurately describe these new findings.	**Results** (pp. 13-14), **Table 3** (p. 14)
**2**	**Conclusion Language**	**Reviewer #5:** Rewrite Conclusion to avoid causal language. Use association-focused wording (e.g., "was associated with," "suggest EI may be an important correlate").	The Conclusion has been thoroughly revised to remove all causal language. We have replaced phrases like "protective factor" and "emerges as a cornerstone" with appropriate cross-sectional phrasing that emphasizes association, not causation, as per the provided examples.	**Conclusion** (p. 23)
**3a**	**Abstract Specificity**	**Editor:** Specify the design precisely and include data-collection dates.	The Abstract has been updated to include the precise study design ("cross-sectional, descriptive correlational") and the data collection period ("February-April 2025").	**Abstract, Methods** (p. 3)

**3b**	**Terminology**	**Editor:** First use "emotional intelligence (EI)", then use "EI" consistently.	The terminology has been standardized throughout the manuscript. The first instance is now "emotional intelligence (EI)" and the abbreviation "EI" is used consistently thereafter.	Throughout manuscript
**3c (i)**	**Methods: Sampling**	**Editor:** Do not call a convenience sample "representative."	The term "representative" has been removed from the manuscript. The sampling method is now accurately described as convenience sampling without claims of representativeness.	**Methods** (p. 6)
**3c (ii)**	**Methods: Hospital Justification**	**Editor:** Justify excluding private hospitals and cite an official source.	We have added a justification for excluding private hospitals, citing the Palestinian Ministry of Health to describe the standardized protocols and similar resource constraints in governmental hospitals.	**Methods** (p. 6) - **Ref. [16]** added
**3c (iii)**	**Methods: Sample Size**	**Editor:** State why the sample was increased to 230.	We have added a sentence explicitly stating that the sample was increased to 230 to account for potential non-responses and dropouts, ensuring adequate statistical power.	**Methods** (p. 7)
**3c (iv)**	**Methods: Experience Criterion**	**Editor:** Justify the ≥6-month experience criterion.	We have added a rationale for the 6-month inclusion criterion, explaining it ensures participants have completed orientation and are familiar with NICU-specific challenges.	**Methods** (p. 7)

**3c (v)**	**Methods: Section Title**	**Editor:** Rename "Instruments" to "Measures".	The section title has been changed from "Instruments" to "Measures".	**Methods** (p. 8)
**3c (vi)**	**Methods: UWES Reliability**	**Editor:** Include UWES reliability in the Measures section.	The reliability (Cronbach's alpha) for the UWES in this study has been added to the Measures section.	**Measures** (p. 9)
**3c (vii)**	**Methods: Effect Size Guidelines**	**Editor:** Cite Cohen's guidelines for interpreting correlation strengths.	We have added a citation to Cohen (1988) for the interpretation of correlation coefficients and added the full reference to the reference list.	**Statistical Analysis** (p. 10) - **Ref. [2]** added
**3d (i)**	**Results: Duplicate Info**	**Editor:** Remove any duplicated response-rate sentence.	The duplicate sentence regarding the response rate has been removed from the Results section. The information is now presented clearly and once.	**Results** (p. 11)
**3d (ii)**	**Results: Regression Table**	**Editor:** In regression table, include standardized β, t, 95% CIs, exact p, and mark significance.	Table 4 has been updated to include the standardized beta coefficients (β), t-values, exact p-values, 95% Confidence Intervals, and significance markers (*).	**Table 4** (p. 15)
**3d (iii)**	**Results: R^2 Interpretation**	**Editor:** Briefly interpret variance explained (R^2/adj-R^2) in plain language.	We have added a plain-language interpretation of the R^2 value in the Results text, stating the predictors "collectively explained 57.4% of the variance."	**Results** (p. 15)
**4**	**Limitations: Bias**	**Reviewer #6:** Expand limitations to name specific biases (e.g., self-selection, non-response, confirmation bias).	The Limitations section has been significantly expanded to explicitly name and discuss potential self-selection bias, non-response bias, and confirmation	**Limitations** (p. 22)

			bias, in addition to the previously mentioned selection bias.	
**-**	**Supplemental File**	**Editor:** Upload SPSS syntax/output as a Supplement.		Uploaded
